# KPNA2 promotes the progression of gastric cancer by regulating the alternative splicing of related genes

**DOI:** 10.1038/s41598-024-66678-7

**Published:** 2024-07-25

**Authors:** Xia Chen, Hui Wei, Ailin Yue, Huiyun Zhang, Ya Zheng, Weiming Sun, Yongning Zhou, Yuping Wang

**Affiliations:** 1 The First Clinical Medical College, Lanzhou University, Lanzhou, 730000 China; 2 Department of Gastroenterology, Key Laboratory for Gastrointestinal Diseases of Gansu Province, The First Hospital of Lanzhou University, Lanzhou, 730000 Gansu Province China; 3 Gansu Province Clinical Research Center for Digestive Diseases, The First Hospital of Lanzhou University, Lanzhou, 730000 China; 4 Department of Endocrinology, The First Hospital of Lanzhou University, Lanzhou, 730000 China

**Keywords:** Alternative splicing, Splicing regulatory sequence, Immunization, KPNA2, Gastric cancer, Gastric cancer, Gastroenterology

## Abstract

RNA-binding proteins (RBPs) play critical roles in genome regulation. In this study, we explored the latent function of KPNA2, which is an essential member of the RBP family, in the regulation of alternative splicing (AS) in gastric cancer (GC). We analyzed the role of KPNA2 in regulating differential expression and AS via RNA sequencing (RNA-seq) and improved RNA immunoprecipitation sequencing (iRIP-seq). Clinical specimens were used to analyze the associations between KPNA2 expression and clinicopathological characteristics. CCK8 assays, transwell assays and wound healing assays were performed to explore the effect of KPNA2/WDR62 on GC cell progression. KPNA2 was shown to be highly expressed in GC cells and tissues and associated with lymph node metastases. KPNA2 promoted the proliferation, migration and invasion of GC cells and primarily regulated exon skipping, alternative 3's splice sites (A3SSs), alternative 5' splice sites (A5SSs), and cassette exons. We further revealed that KPNA2 participated in biological processes related to cell proliferation, and the immune response in GC via the regulation of transcription. In addition, KPNA2 preferentially bound to intron regions. Notably, KPNA2 regulated the A3SS AS mode of WDR62, and upregulation of WDR62 reversed the KPNA2 downregulation-induced inhibition of GC cell proliferation, migration and invasion. Finally, we discovered that the AS of immune-related molecules could be regulated by KPNA2. Overall, our results demonstrated for the first time that KPNA2 functions as an oncogenic splicing factor in GC that regulated the AS and differential expression of GC-related genes, and KPNA2 may be a potential target for GC treatment.

## Introduction

The human genome sequence consists of exons and introns and is discontinuous. Alternative splicing (AS) is the process by which introns are removed from pre-mRNAs and exons are linked to produce mature mRNAs^1^. More than 95% of human genes undergo AS, resulting in the production of multiple mRNAs and protein isoforms from a single gene^2^. Increasing research has shown that AS can produce new markers by regulating RNA transcription in tumors^3–5^. It has recently been reported that splicing may be closely associated with tumorigenesis and that abnormal changes in AS possibly interfere with tumor progression and protein interaction mechanisms during tumor development^6,7^. In recent years, an increasing number of studies have revealed the breadth of control of AS in the immune system, which has increased the understanding of the mechanisms that regulate these processes and the extent to which AS influences the lymphocyte responses to immune challenges^8–10^. As of 2020, there were 1.09 million new cases of gastric cancer (GC), and GC caused 0.77 million deaths; GC was one of the leading causes of cancer, ranking 4th in mortality and 5th in incidence worldwide^11^. Multiple factors, ranging from genetic aberrations to environmental factors, play a role in the occurrence and progression of GC. AS is known to be closely related to in the metastasis, drug resistance, and prognosis of GC^12–14^. Emerging studies, such as those involving high-throughput sequencing, have improved our understanding of the pathogenesis of GC in a molecular context^15^. The development of novel validated biomarkers and the identification of the most plausible mechanisms for predicting progression could increase the specificity and precision of GC prevention and risk prediction^16^.

KPNA2, which is an RNA-binding protein (RBP), can interact with nuclear localization sequences (NLSs) to participate in nuclear transport and transcriptional regulation^17–19^. It has been shown that RBPs usually bound to sequences in the untranslated region of mRNA and regulated the stability and translation efficiency of mRNA, which in turn affected gene expression^20^. KPNA2 has been confirmed to be upregulated in some solid tumors^21–25^. Many viral proteins may target KPNA2 and inhibit its function as a defense strategy against the host immune response^26^. Additionally, KPNA2 can moderate key molecular pathways at the transcriptional, posttranscriptional, and posttranslational levels. Our work revealed that KPNA2 promoted the migration and invasion of GC cells. Overexpression of KPNA2 has been associated with poor patient prognosis, and inhibition of KPNA2 expression has the potential to arrest the migration and invasion of GC cells^27,28^. It has been reported that even slight changes in the activity or abundance of individual RBPs or kernel spliceosomal proteins may lead to major changes in the splicing of specific transcripts^29^. Nevertheless, whether KPNA2 acts as an RBP in GC, affecting gene expression and AS, as well as its function and molecular mechanism of action in GC are unclear.

**Figure 1:**
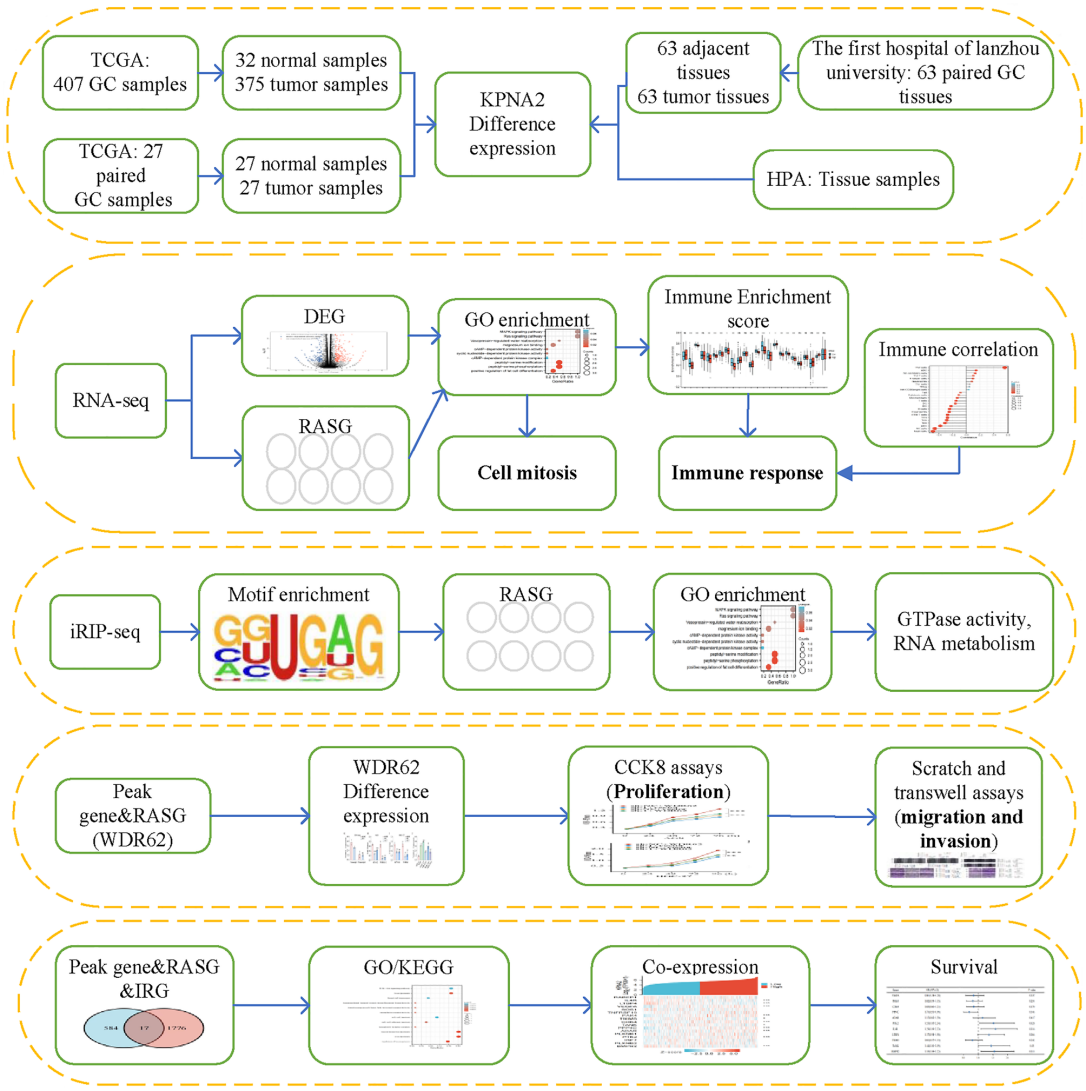
Study flowchart.

We designed a synthetic analysis scheme for this study (Fig. 1). KPNA2 mRNA was aberrantly upregulated in GC cells and tissues. Additionally, KPNA2 may regulate GC-related gene expression and AS to participate in biological processes, such as the immune response and cell adhesion, as determined by high-throughput RNA sequencing (RNA-seq) and improved RNA immunoprecipitation sequencing (iRIP-seq). KPNA2 can also regulate the AS of immune-related genes (IRGs). Interestingly, we observed that KPNA2 promoted the proliferation and metastasis of GC cells, which was reversed by WDR62 transcript 1.

## Materials and methods

### KPNA2 expression in digestive system cancers

A total of 1650 genes were identified by searching the GeneCards database (https://www.genecards.org/) with “GC” and “RNA binding” as the keywords. A total of 619 GC samples were divided into carcinoma tissue samples and adjacent tissue samples (408 carcinoma tissue samples and 211 adjacent tissue samples) in the GEPIA (http://gepia.cancer-pku.cn/) database, and these samples were used for differential expression analysis of the genes (false discovery rate (FDR) ≤ 0.01 and |log2fold change (log2FC)|≥ 2). Ultimately, 843 differentially expressed genes (DEGs) were identified.

The GEPIA database (http://gepia.cancer-pku.cn/) was used to analyze KPNA2 mRNA expression in cancers of the digestive system, with the inclusion criteria of log2FC > 2 and *p* < 0.05. The raw data (expression profiles) for human GC were downloaded from The Cancer Genome Atlas (TCGA) database (https://portal.gdc.cancer.gov). The existing mRNA sequencing (mRNA-seq) data of 375 GC samples (including 27 paired GC samples) were obtained from the TCGA database. R software (3.6.3) was used to visualize differences in the expression of KPNA2 mRNA between carcinoma tissues and adjacent tissues from the TCGA database using the ggplot2 package (3.3.3). The Human Protein Atlas (HPA) (https://www.proteinalas.org/) was used to determine KPNA2 protein expression in GC.

### RNA-seq

For every sample, RNA-seq libraries were prepared using the KAPA Stranded mRNA-Seq Kit for Illumina® platform (#KK8544) with 1 μg of gross RNA. 150 nt of paired-end sequencing was performed on the libraries by the Illumina Novaseq 6000 system.

### RNA-Seq raw data clean and alignment

Discard raw reads with more than 2-N bases. Then, the raw sequencing data was trimmed by the FASTX-Toolkit (version 0.0.13). Next, clean reads were adjusted to the GRch38 genome using tophat2, which allowed for four mismatches^30^. Universally mapped reads were taken to calculate gene reads and conduct FPKM calculations.

### DEGs analysis

The R Bioconductor package edgeR was applied to screen for DEGs regulated by KPNA2. The cutoff criteria for DEGs were framed as fold change > 2 or < 0.5 and FDR < 0.05.

### Data analysis

To assess RBP-regulated alternative splicing events (ASEs), the student's t-test was applied to estimate the significance of the change in ratio of AS events. *p* < 0.05 was considered significant for ASEs. The KPNA2 binding regions in the genome were determined by the "ABLIRC" strategy^31^. All observed heights of peaks above the random maximum peak were selected (*p* < 0.05). Input and IP samples were analyzed separately by simulation, with the subsequent removal of IP peaks overlapping with the input peaks. The IP sample target genes were eventually confirmed by peaks, and the HOMER software was used to call the IP protein binding motifs^32^.

### Analysis of KPNA2 expression and immune infiltration in GC

To explore the correlations between KPNA2 copy number and tumor-infiltrating immune cells in GC, we used the TIMER (http://timer.cistrome.org/) database, which is an online tool for analyzing the correlations between variables and immune infiltration levels across cancer types. The “GSVA” package (1.34.0) and the ssGSEA algorithm were used to determine the correlation between KPNA2 expression and 24 immune cell types^33,34^ in the GC dataset from the TCGA database. The Shapiro‒Wilk normality test was used to explore the correlation between KPNA2 expression and Th2 cell marker expression.

### Analysis of IRG AS

We extracted 1793 unique IRGs from the Immunology Database and Analysis Portal database (ImmPort) (https://www.immport.org/shared/home). A Venn diagram was generated to determine the intersection of genes that underwent AS and IRGs that underwent regulated AS (RASG). The clusterProfiler package (3.14.3) was used to conduct the GO/KEGG enrichment analysis. The org.Hs.eg.db package (3.10.0) was used for ID conversion, and the survival package (3.2.10) was used for the statistical analysis of survival data.

### Patient and tissue samples

Sixty-three GC samples and adjacent tissue samples were obtained from the First Hospital of Lanzhou University. All the samples were obtained from patients who did not undergo preoperative radiation therapy and had a diagnosis of GC that was confirmed by histology. Informed consent forms were signed by all the participants, and the study was approved by the Ethics Committee of the First Hospital of Lanzhou University for the purpose of the present study (LDYYLL2023-158). All the methods were performed in accordance with the relevant guidelines and regulations.

### Cell culture

The GSE-1, MKN-45, AGS, HGC-27, MKN-28, and MGC-803 cell lines (Cellcook, China) were cultured at 37 °C with 5% CO_2_ in complete medium supplemented with 10% serum (ABW, URU).

### Quantitative RT‒qPCR

DNA was removed from total RNA with RQ1 DNase (Promega). The quantity and quality of the purified RNA were determined by using a Nanodrop One (Thermo Fisher, USA). cDNA synthesis was conducted using standard procedures, followed by RT‒qPCR with Hieff qPCR SYBR® Green Master Mix (YEASEN, China). The primers that were used for qRT‒PCR are shown in Supplemental Table 1.

**Table 1:** High-quality clean reads of iRIP-seq.

Sample ID	RAW	Clean	Raw base	Clean base	Unique Tag	Q20 (%)	Q30 (%)	DUP (%)
KPNA2_IP_1	41648652	39899321 (95.80%)	6.25G	5.41G (86.62%)	9450813 (23.69%)	98.25	94.89	93.27
KPNA2_IP_2	40714564	39044923 (95.90%)	6.11G	5.30G (86.86%)	8970225 (22.97%)	98.18	94.72	93.61
KPNA2_input_1	70240752	69171773 (98.48%)	10.54G	7.78G (73.86%)	13739402 (19.86%)	98.64	95.54	95.98
KPNA2_input_2	54821866	53893644 (98.31%)	8.22G	5.86 (71.29%)	11426018 (21.20%)	98.63	95.52	95.64

(1) RAW: Number of raw sequences transformed from primary image data obtained by base-call sequencing; (2) Clean: Stripping adapter sequences from raw reads, the number of valid sequences acquired after low quality bases for subsequent analysis; (3) Raw base: Based on the length and number of the raw reads, calculate the number of bases they contained, in G; (4) Clean base: Depending on the number and length of the clean reads, the number of bases they contain was calculated, in G; (5) Ratio of the number of non-repeat reads to their clean reads.; (6) Q20: The proportion of bases with sequencing error rates below 1%.; (7) Q30: The proportion of bases with sequencing error rates below 0.1%; (8) DUP: Duplication level. Ratio of duplicate reads to total reads.

### Western blotting

The proteins of GC cells and tissues were extracted by lysing the cells with RIPA buffer (Solarbio, China) supplemented with protease inhibitors (Solarbio, China) according to the manufacturer's instructions. Equal amounts of proteins were separated by SDS‒PAGE and transferred to PVDF membranes for Western blotting. The membranes were incubated with Tris buffer supplemented with 0. 05% Tween (TBST) and 5% skim milk for 1 h at room temperature. The primary antibodies that were used were anti-KPNA2 (1:1000, Proteintech, China) and anti-β-actin (1:1000, Proteintech, China) antibodies; these antibodies were incubated with the membranes overnight at 4 °C. After washing three times with TBST, the membrane was incubated with the secondary antibodies at room temperature for 1 h, and a chemiluminescent solution was used for imaging (Monad, China).

### Cell transfection

The construction of WDR62 overexpression plasmid was based on WDR62 transcript 1 (NM_001083961, Related to ENST00000401500.6). The shKPNA2 (sh-1 and sh-2) and WDR62 plasmids were purchased from GeneChem (Shanghai, China). siRNA-KPNA2 targeting sequences (GenePharma, China) were as follows: sh-1: 5′- GCAUCAUGAUGAUCCAGAATT -3′ (sense), 5′-UUCUGGAUCAUCAUGAUGCTT-3′ (antisense); sh-2: 5′- GGAGCUUCUGAAUUGCCAATT-3′ (sense), 5′- UUGGCAAUUCAGAAGCUCCTT -3′ (antisense). The cells were transfected with Lipofectamine 2000 (Invitrogen, USA) according to the manufacturer's protocol.

### CCK8 cell proliferation assay

Cell viability was tested by CCK-8 assay, 5000 GC cells were inoculated onto 96-well plates and cultured. Then, commercial CCK-8 solution was added to each well and incubated for one hour. The absorbance at 450 nm was measured at 0 h, 24 h, 48 h, 72 h and 96 h with an enzyme marker, respectively.

### Transwell and wound healing assays

Cell migration and invasion assays were carried out in Transwell chambers (Corning, USA). Cells were collected 24 h after transfection. The cells were suspended (1 × 10^5^ cells) in 100 μl of serum-free medium and seeded in the top chamber that had been precoated with matrix gel (invasion), and 500 μl of complete medium supplemented with 20% serum was added to the bottom chamber. After incubating for 24 h, a cotton swab was used to remove the cells that did not cross the membrane. The cells on the lower side of the membrane were fixed with 4% paraformaldehyde and stained with a 0.1% solution of crystal violet. A digital microscope (Olympus, Japan) was used to image invading or migrating cells.

For the wound healing assay (2 × 10^5^ cells), cell monolayers were scratched with the tip of a p200 pipette. Images were taken with a digital microscope (Olympus, Japan) at 0 h, 12 h and 24 h. The wound healing rate was calculated by repeating the experiment three times and taking the average value.

### Ethical approval and consent to participate

All participants signed an informed consent form, and the Ethics Committee of the first hospital of Lanzhou university approved the study (LDYYLL2023-158).

## Results

### Differential expression of KPNA2

**Figure 2:**
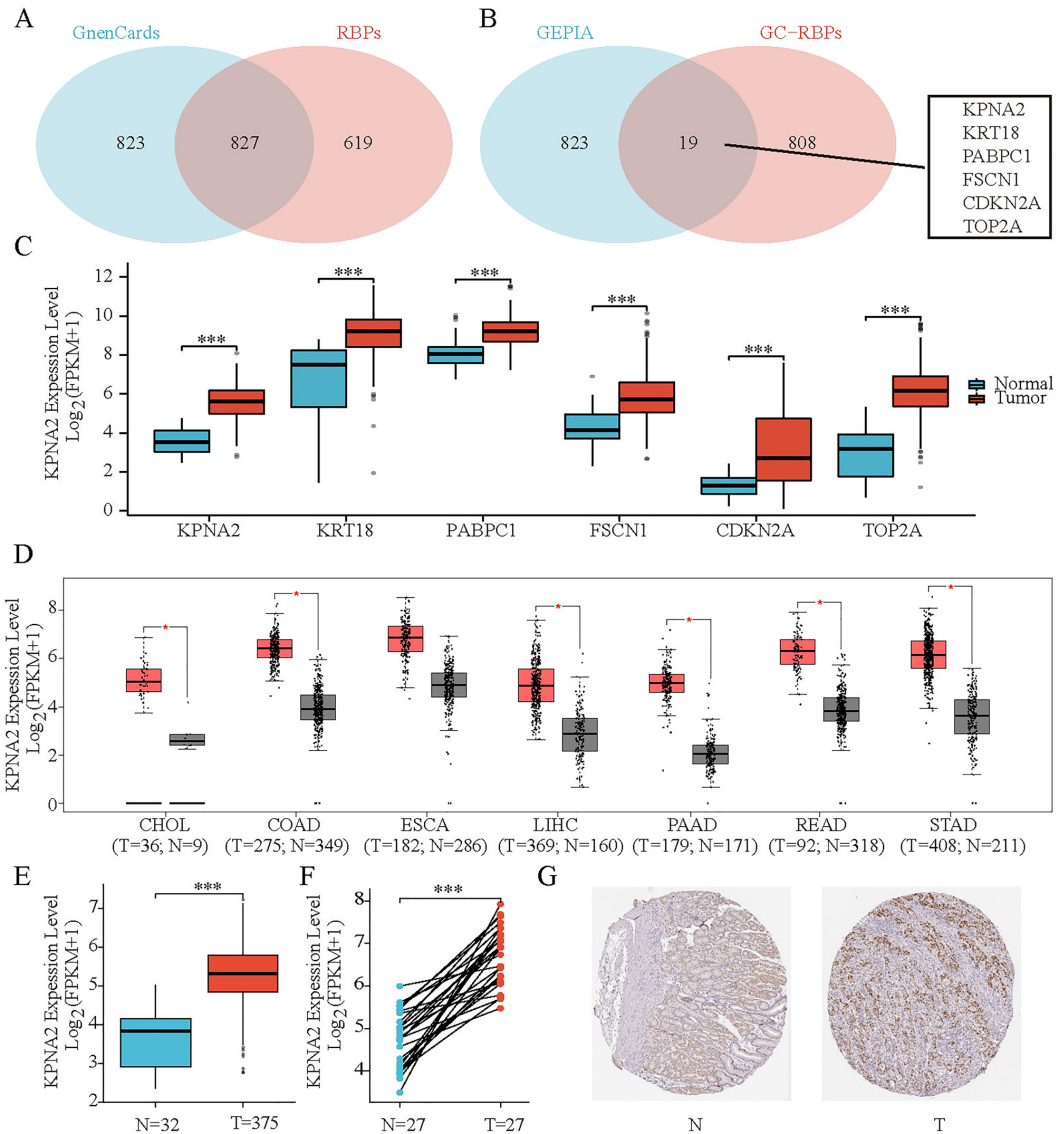
Differential expression of KPNA2. (**A**,**B**) Venn diagram. (**C**) Expression of several RBPs in GC. (**D**) Comparison of KPNA2 expression in digestive system cancer samples and normal tissue samples from GEPIA. (**E**,**F**) KPNA2 was overexpressed in GC in unpaired and paired TCGA samples. (**G**) Elevated protein expression of KPNA2 in GC from the HPA. *p < 0.05, **p < 0.01, ***p < 0.001.

We first examined the intersection of 1650 GC-related genes in GeneCards with 1455 RBPs, and we identified 827 GC-related RBPs (Fig. 2A). Subsequently, 19 differentially expressed RBPs were identified from 843 DEGs (Fig. 2B). Various RBPs, such as KPNA2, KRT18, PABPC1, FSCN1, CDKN2A and TOP2A, were found to be highly expressed in GC (Fig. 2C). Using GC and the aforementioned RBPs as keywords, a PubMed literature search (https://pubmed.ncbi.nlm.nih.gov/) was performed and revealed that the function of KPNA2 in GC has not been well studied. In this study, according to the GEPIA database, KPNA2 expression was increased in cancers of the digestive system (Fig. 2D). The mRNA expression of KPNA2 was significantly upregulated in GC patients according to the TCGA database (Fig. 2E,F). Additionally, the downregulation of KPNA2 was observed by immunohistochemistry (IHC) of samples that were acquired from the HPA database (Fig. 2G).

### KPNA2 regulated gene expression in AGS cells

**Figure 3:**
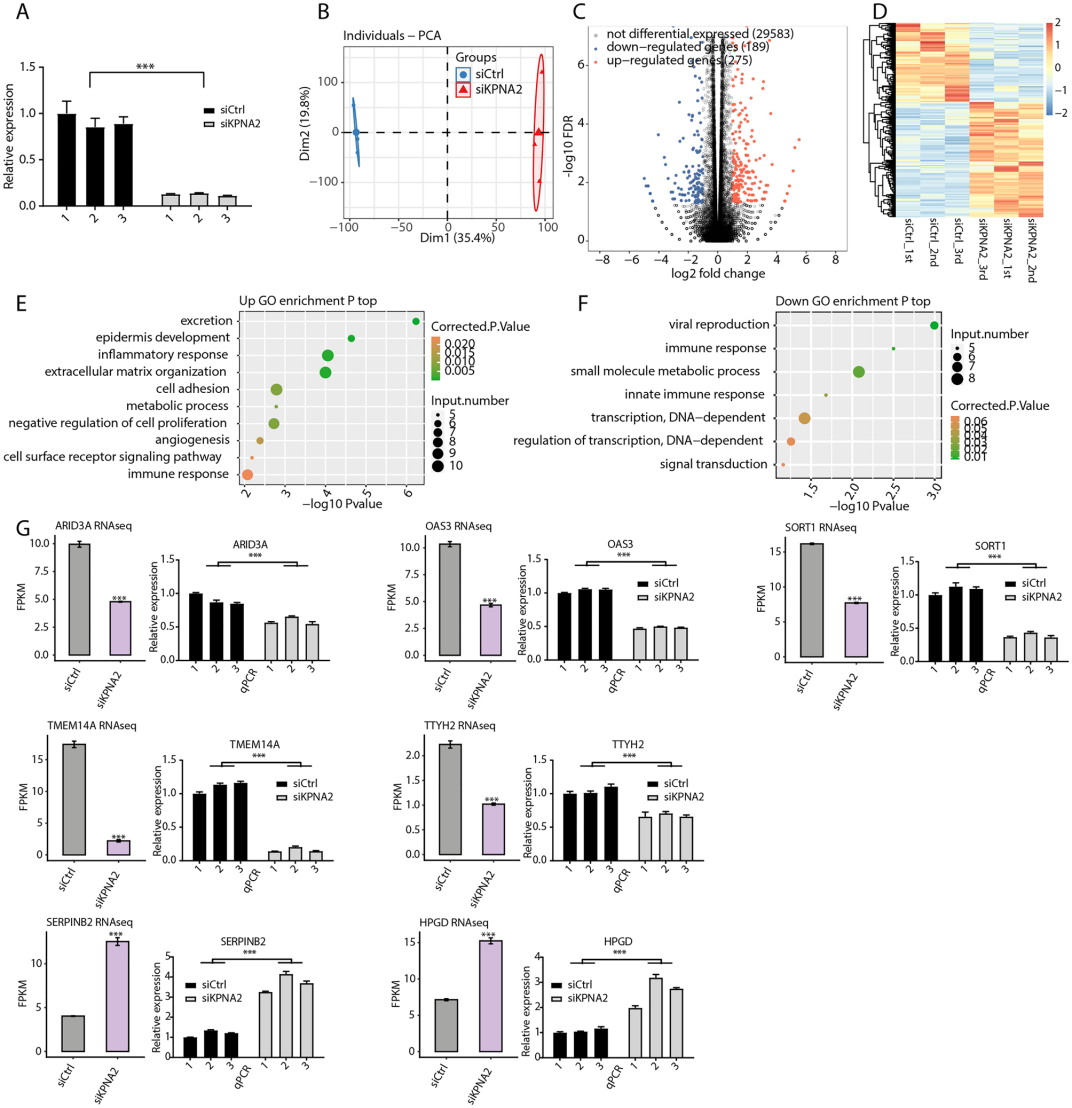
KPNA2 regulated gene expression in AGS cells. (**A**) KPNA2 expression was quantified by RT‒qPCR. Error bars represent the mean ± SEM. (**B**) PCA of two groups of samples based on the normalized gene expression level. (**C**) Identification of KPNA2-regulated genes. (**D**) Hierarchical clustering of DEGs in control and KPNA2-knockdown samples. (**E**,**F**) The top ten representative GO biological processes of up- or downregulated genes. (**G**) RNA-seq and RT‒qPCR demonstrated that some differentially expressed genes were regulated by KPNA2. The error bars indicate the means ± SEMs. ***p < 0.001.

We used RNA-seq to explore the functions of latent KPNA2 in AGS cells. We first knocked down KPNA2 in AGS cells via siRNA transfection (Fig. 3A). Principal component analysis (PCA) revealed that the FPKMs of all the detected genes were clustered within the group, with good intragroup repeatability and similar sample data. There was good differentiation between the siCtrl and siKPNA2 groups (Fig. 3B). Interestingly, silencing of KPNA2 resulted in the upregulation of 275 genes and the downregulation of 189 genes (FC ≥ 2 or ≤ 0.5, FDR < 0.5) (Fig. 3C). Details about these DEGs can be found in Supplementary Table 2. Heatmap analysis of DEG expression profiles based on RNA-seq data revealed a high degree of consistency in KPNA2-regulated transcription among the three sets (Fig. 3D). Next, we identified several pathways that included upregulated genes annotated with categories including epidermal development, inflammatory response, cell adhesion, negative regulation of cell proliferation, and immune response (Fig. 3E). Additionally, GO analysis revealed several biological processes, including immune response, small molecule metabolic process, innate immune response, and transcription, which were significantly enriched for the downregulated genes (Fig. 3F). According to the gene expression levels, the significantly upregulated DEGs in the siKPNA2_1st group were arranged in descending order, as were the significantly downregulated DEGs in the siCtrl_1st group. The top 30 up- and downregulated DEGs are shown in Supplementary Tables 3 and 4, respectively. We found that ARID3A, OAS3, SORT1, TMEM14A, TTYH2, HPGD, and SERPINB2 were associated with cancers^35–41^. The hypoexpression of KPNA2 induced downregulation of ARID3A, OAS3, SORT1, TMEM14A, and TTYH2 and the upregulation of HPGD and SERPINB2, and these findings were further verified by RT‒qPCR (Fig. 3G).

### KPNA2 regulated gene AS in AGS cells

**Figure 4:**
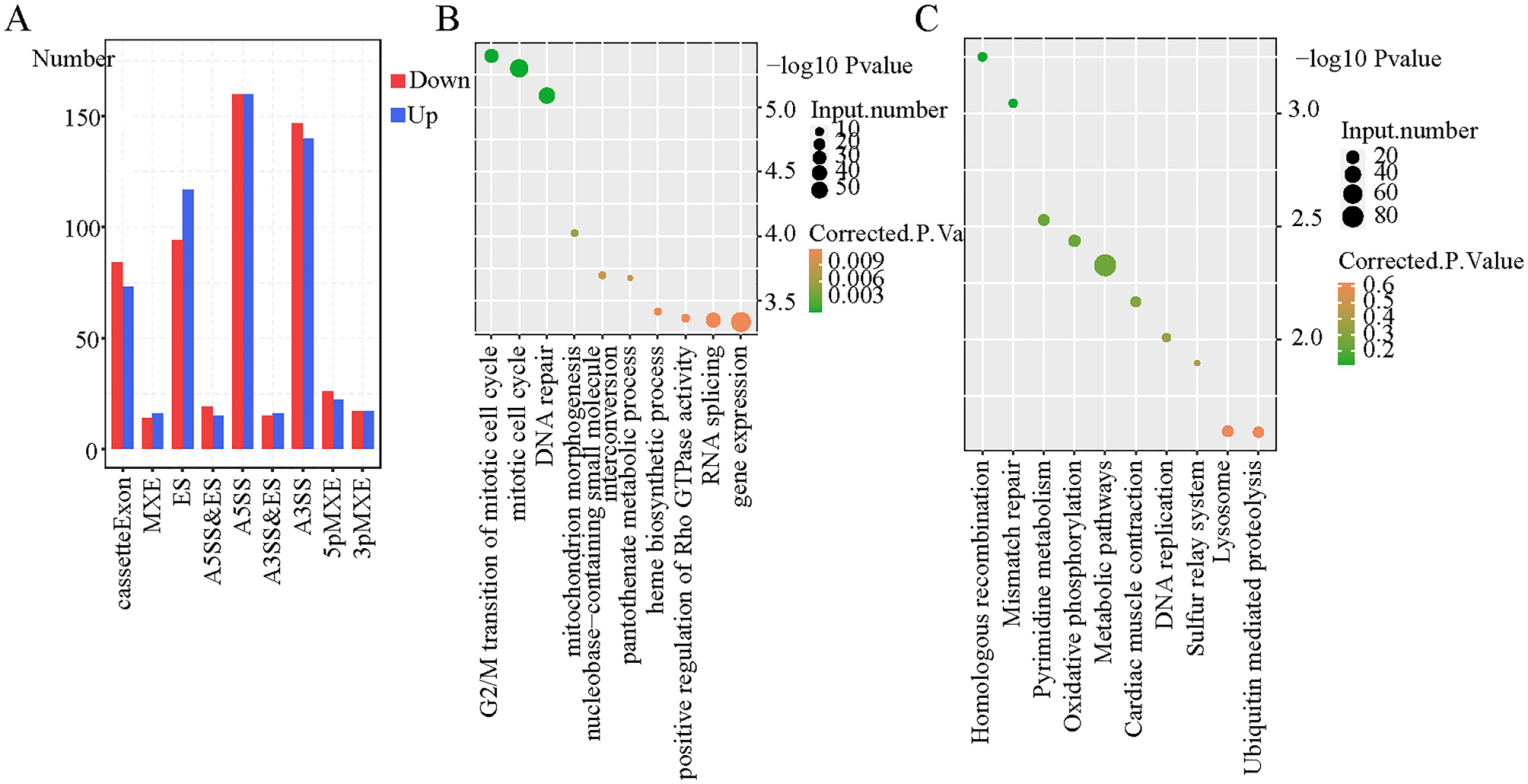
KPNA2 regulated gene AS in AGS cells. (**A**) Frequency distribution of KPNA2-regulated ASEs. (**B**,**C**) GO and KEGG biological processes of RASGs. *p < 0.05, **p < 0.01, ***p < 0.001.

It is unclear whether KPNA2, which is an RBP, affects gene AS in GC. Therefore, we used RNA-seq to determine whether KPNA2 regulates AS (RASE) in AGS cells. A total of 1560 ASEs were altered after KPNA2 was silenced (Supplementary Table 5). The main RASEs were the alternative 5′ splice site (A5SS), alternative 3′ splice site (A3SS), exon skipping (ES), and cassette exon, in addition to mutually exclusive 3′UTRs (3pMXE), mutually exclusive 5′UTRs (5pMXE), A5SS and ES, and mutually exclusive exons (MXE) (Fig. 4A). Taken together, these results indicated that the A5SS, A3SS, ES, and cassette exons were the main ASEs that were regulated by KPNA2 and that KPNA2 had a comprehensive regulatory effect on AGS cells. A total of 1241 RASGs remained after KPNA2 silencing. GO analysis was performed on these RASGs, which were enriched in biological processes such as the G2/M transition of the mitotic cell cycle, mitotic cell cycle, and DNA repair (Fig. 4B). According to KEGG enrichment analysis, these RASGs were enriched in the pathways of homologous recombination, mismatch repair, and pyrimidine metabolism (Fig. 4C).

### KPNA2 bound to mRNAs associated with GC in AGS cells

The RBPs are assessed based on their capacity to bind to RNA and the amount of these proteins that can be effectively recovered. To gain insight into the specificity of KPNA2 pull-down experiments, we performed iRIP-seq to identify the mRNAs that bound to KPNA2. Two replicate experiments were conducted to ensure the reliability of the results. iRIP-seq showed good quality control and reproducibility in the IP group (Table 1). In summary, these results demonstrated that KPNA2 can specifically bind to mRNA.

**Figure 5:**
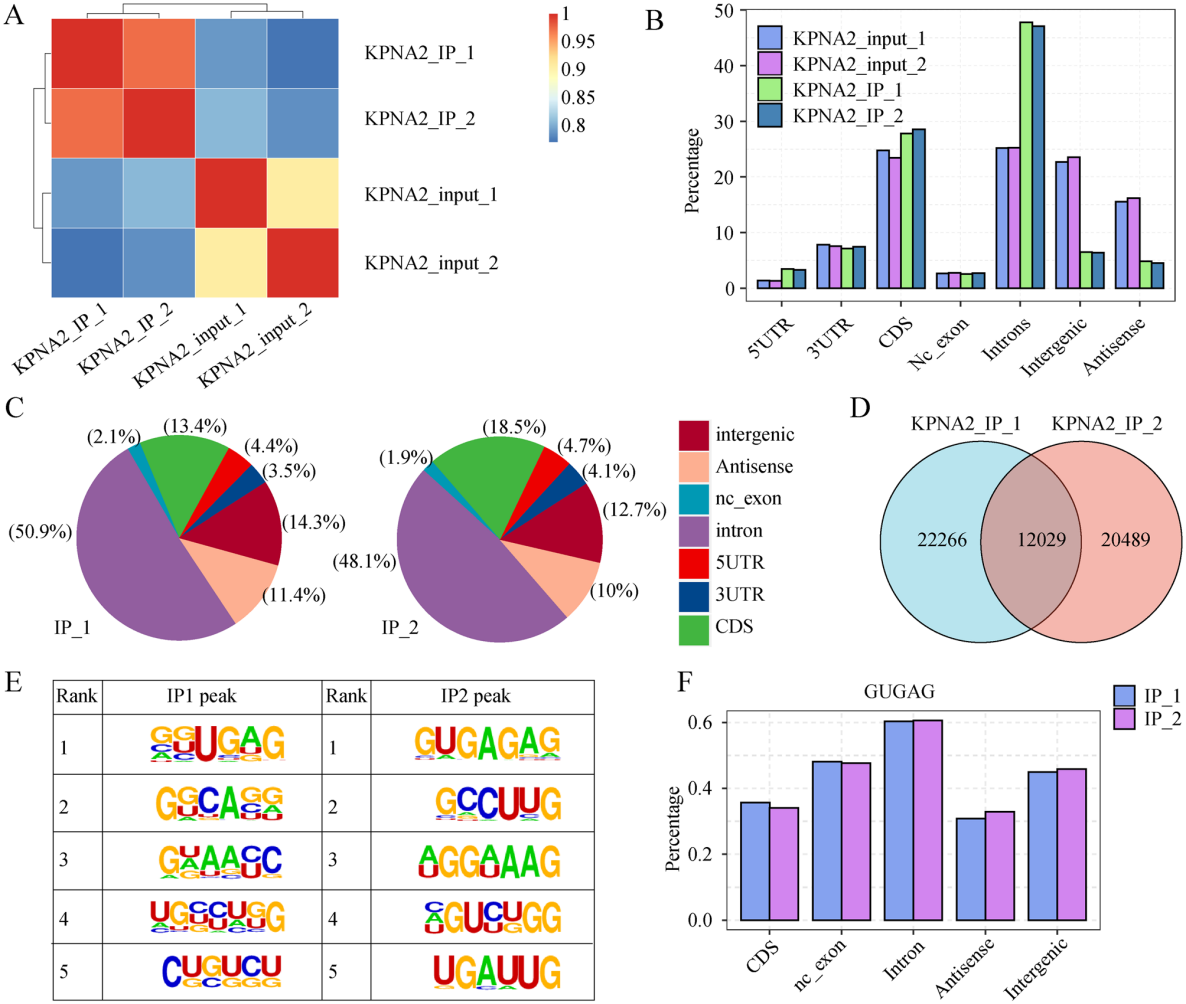
KPNA2 bound to mRNAs associated with GC in AGS cells. (**A**) Heatmap showing the correlation between the IP and input groups. (**B**) Bar plot showing the read distribution across the reference genome. (**C**) Pie charts of KPNA2-bound peaks across the reference genome. (**D**) Venn diagram showing the overlap of KPNA2 binding peaks identified in the two trials. (**E**) Motif enrichment of KPNA2-bound peaks by HOMER. (**F**) Bar plot showing the distribution of peaks containing the GUGAG motif.

As shown in Fig. 5A and Fig. S1A, the correlations of the samples were 0.711 and 0.706, respectively, indicating that the reads were significantly enriched in the IP group relative to the input group. The genomic distribution of the reads showed that the KPNA2-IP reads were enriched in the 5′UTR, CDS, and intron regions compared to those in the input group (Fig. 5B) and most significantly enriched in the introns (Fig. 5C); this result suggested that KPNA2 may bind to these regions to regulate transcript stability, AS, and translation processes. A total of 12,029 KPNA2-binding regions were identified in the two replicate trials using the Ablife method for peak calling of the binding regions (Fig. 5D). Notably, the binding motifs (GUGAG) of KPNA2 that were identified in the two replicate trials were consistent (Fig. 5E). The GUGAG motif was also mainly distributed in the intron region (Fig. 5F). The genes associated with the KPNA2 binding peak in the KPNA2-IP group are shown in Supplementary Table 6 and Supplementary Table 7. GO analysis revealed that genes that were associated with KPNA2 binding peaks that were observed in both trials were enriched for protein phosphorylation, gene expression, mitotic cell cycle, and other biological processes (Fig. S1B), which provides a basis for future studies on the integrated regulation of KPNA2-RNA interactions in AGS cells.

### KPNA2 regulated pre-RNA AS by binding to pre-mRNAs

**Figure 6:**
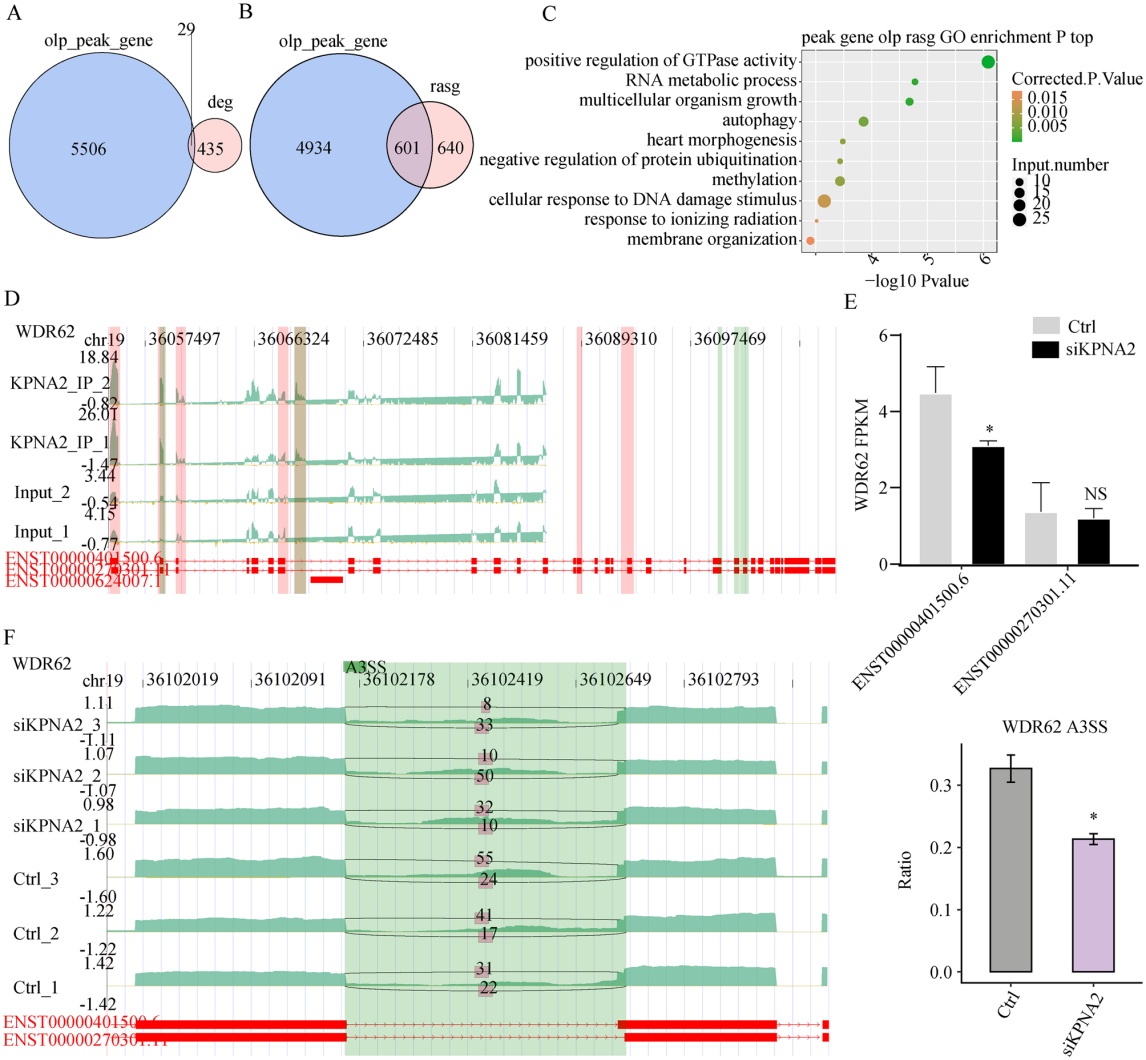
Integrated analysis of KPNA2-bound genes and RASE in response to KPNA2 knockdown. (**A**) Venn diagram showing the overlap of KPNA2-bound peaks and KPNA2-regulated DEGs. (**B**) The overlap of KPNA2-binding genes with KPNA2-regulated RASGs. (**C**) The top ten representative GO biological processes of the overlapping genes. (**D**) KPNA2 binds to WDR62 mRNA. (**E**) Changes in the expression levels of the WDR62 transcript. (**F**) KPNA2 regulated the AS of WDR62. *p < 0.05, **p < 0.01, ***p < 0.001.

Twenty-nine genes were found to have mRNAs or antisense RNAs with KPNA2 binding sites (Fig. 6A). Because KPNA2, which is a splicing factor, binds to RNA and affects AS, our aim was to investigate the effect of KPNA2 binding to pre-mRNAs on AS. Overlay analysis was performed using the 1241 genes that corresponded to 1560 significantly different AS events that were identified in the RNA-seq data and the 5535 genes where the binding peaks appeared in both trials of iRIP-seq. The results revealed that KPNA2 may bind 601 pre-mRNAs to regulate AS (Fig. 6B), and detailed information on the 601 genes is provided in Supplementary Table 8. A total of 601 genes were enriched for biological processes, such as positive regulation of GTPase activity, RNA metabolic processes, methylation, and autophagy (Fig. 6C).

We analyzed ASEs that significantly changed according to their ratios between the experimental and control groups. The iRIP-seq results showed that KPNA2 could bind to WDR62 pre-mRNA (binding motif: GUGAG) (Fig. 6D), and the RNA-seq results showed that after KPNA2 was downregulated, the expression of WDR62 (ENST00000401500.6) decreased (*p* = 0.027), while the expression of the ENST00000270301.11 transcript did not significantly change (*p* = 0.726) (Fig. 6E). Furthermore, the A3SS AS mode of the WDR62 pre-RNA was reduced (*p* < 0.05, Fig. 6F).

### Correlation analysis of KPNA2 expression and immune infiltration

**Figure 7:**
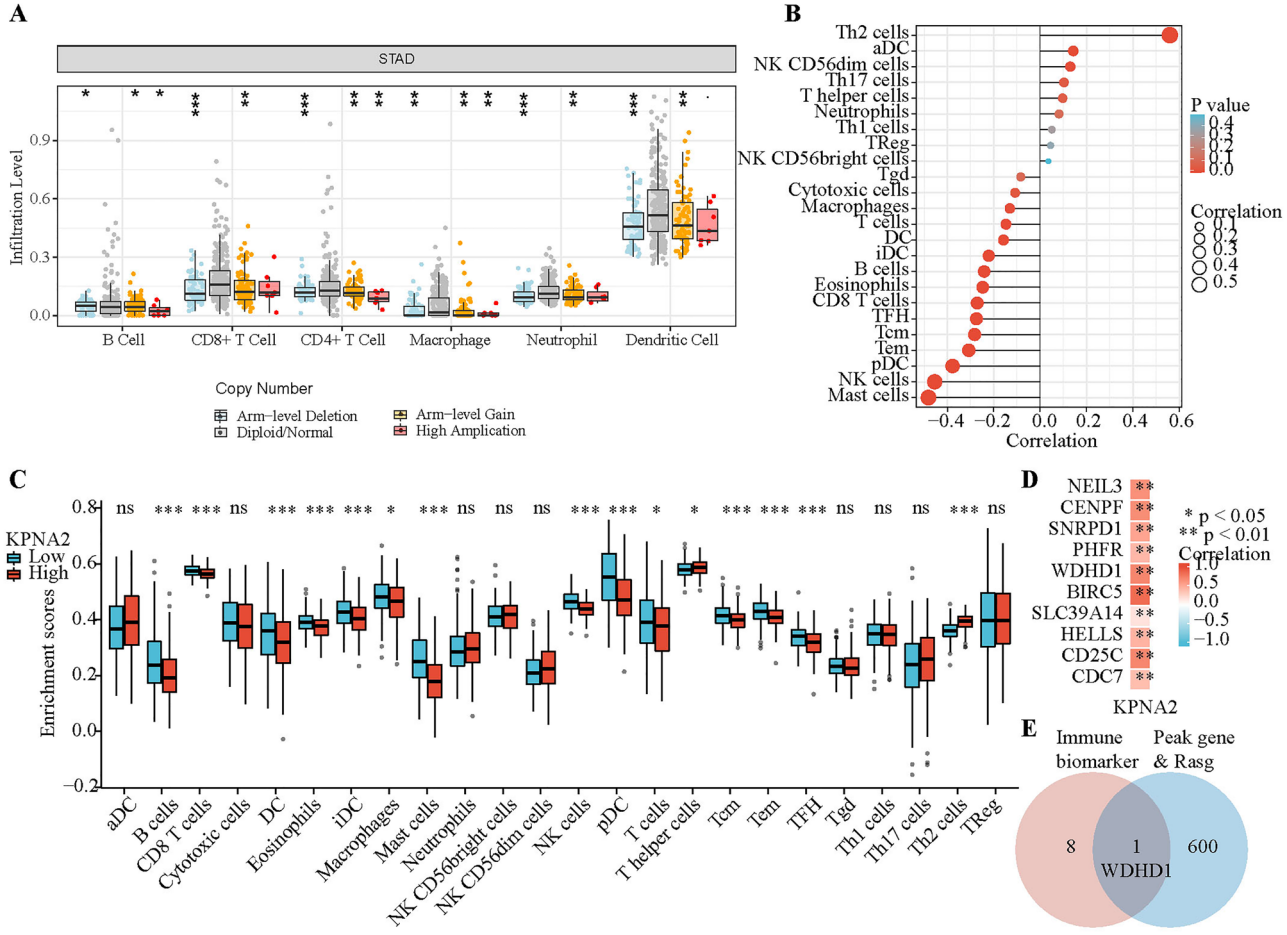
KPNA2 was associated with immune infiltration in GC. (**A**) Relationship between KPNA2 gene copy number and the level of immune cell infiltration in the GC cohort. (**B**) Correlation analysis of KPNA2 expression and immune infiltration in GC. (**C**) Enrichment scores of immune cells in the high and low KPNA2 expression groups. (**D**) Association between KPNA2 and gene markers of Th2 cells. (E) Venn diagram. *p < 0.05, **p < 0.01, ***p < 0.001.

Enrichment analysis revealed that the DEGs that are regulated by KPNA2 were closely related to immunity. As immune cells in the tumor microenvironment (TME) impact patient prognosis, exploring the correlation between KPNA2 expression and immune infiltration is a valuable area of research. First, KPNA2 gene copy numbers seemed to be related to the infiltration of several immune cell types, including neutrophils, B cells, CD4 + T cells, CD8 + T cells, macrophages, and dendritic cells (DCs), in GC according to the “SNCA” plate of TIMER (Fig. 7A). Additionally, we analyzed the correlation between the level of infiltration of each immune cell subset and expression of KPNA2 in patients with GC from the TCGA database (Fig. 7B and Table 2). To determine whether KPNA2 expression correlated with the level of immune infiltration in GCs, we determined the immune cell enrichment scores of high- and low-KPNA2 expression groups (Fig. 7C). The intersection of these two analyses revealed that high KPNA2 expression was correlated with increased infiltration of Th2 cells and reduced infiltration of B cells, CD8 + T cells, DCs, eosinophils, immature DCs, macrophages, mast cells, plasmacytoid DCs, T cells, T central memory cells, T effector memory cells, and T follicular helper cells in GC. The correlation coefficient of the Th2 cell infiltration level with KPNA2 expression was the highest among all the immune cells. Thus, we further explored the association between KPNA2 and markers of Th2 cells^34^. Interestingly, KPNA2 expression was distinctively and positively correlated with the expression of the Th2 cell markers NEIL3, CENPF, SNRPD1, PHFR, WDHD1, BIRC5, SLC39A14, HELLS, CD25C and CDC7 in GC (Fig. 7D). We then screened WDHD1 from among the KPNA2-regulated genes and found that KPNA2 can regulate WDHD1 to mediate the A3SS form of AS (Fig. 7E and Supplementary Table 9).

**Table 2:** Correlations between KPNA2 and immune cells in GC from the TCGA database.

Cell type	Cor	*p*
DC	− 0.158	**
B cell	− 0.241	***
CD8 T cell	− 0.271	**8
Cytotoxic cell	− 0.108	*
aDC	0.142	**
Eosinophil	− 0.248	***
iDC	− 0.222	***
Macrophage	− 0.131	*
Mast cell	− 0.481	***
Neutrophil	0.081	0.117
NK CD56bright cell	0.035	0.495
NK CD56dim cell	0.129	*
NK cell	− 0.455	***
pDC	− 0.378	***
T cell	− 0.148	**
T helper cell	0.097	0.061
Tcm	− 0.283	***
Tem	− 0.308	***
TFH	− 0.275	***
Tgd	− 0.084	0.105
Th1 cell	0.050	0.334
Th17 cell	0.102	*
Th2 cell	0.558	***
Treg	0.045	0.387

DC, dendritic cell; aDC, activated DC; iDC, immature DC; NK, natural killer; pDC, plasmacytoid DC; Tcm, T central memory; Tem, T effector memory; TFH, T follicular helper; Tgd, T gamma delta; Th1, T helper 1; Th17, T helper 17; Th2, T helper 2; Treg, regulatory T cell. **p < 0.05, **p < 0.01, ***p < 0.001.

### KPNA2 may regulate the AS of IRGs

**Figure 8:**
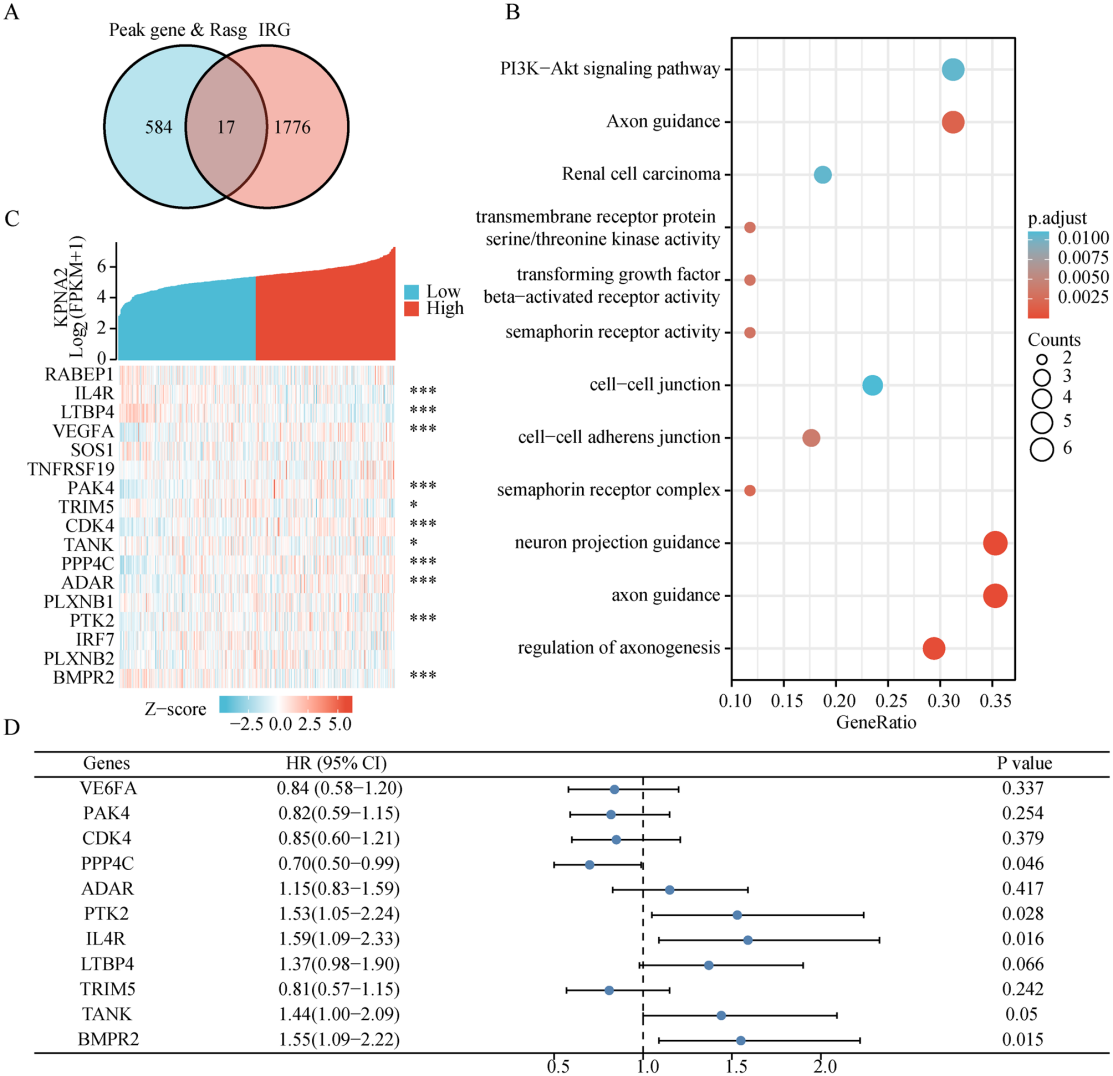
KPNA2 regulated the AS of IRGs. (**A**) Venn diagram showing the overlap of Peak gene & RASG and IRGs. (**B**) GO/KEGG enrichment analysis of IPRG. (**C**) Coexpression analysis of KPNA2 and IPRG. (**D**) Forest plot demonstrating the overall survival analysis of Co-IPRG in GC.

Since the first immune checkpoint inhibitor was approved by the US FDA in 2011, we have witnessed an increase in advances in the field of cancer immunotherapy^42^. Hence, we aimed to further explore the relevance of AS to immunity. We extracted 17 KPNA2-binding IRGs from the peak gene and Rasg (IPRG) (Fig. 8A). KPNA2 could regulate these 17 genes to undergo various forms of AS (RABEP1: ES; PAK4: A5SS; TRIM5: A3SS, etc.), and the details were presented in the Supplementary Table 10. Subsequent GO-KEGG enrichment revealed enrichment of the PI3K-Akt signaling pathway and transmembrane receptor protein serine/threonine kinase activity (Fig. 8B). We examined the pattern of KPNA2 co-expression with these 17 genes. The red dots indicate genes with a positive association with KPNA2, whereas the green dots indicate genes with a negative association with KPNA2 (Fig. 8C). Moreover, we found that GC patients with high expression of PPP4C (hazard ratio [HR] 0.70, 95% confidence interval [CI] 0.50–0.99, *p* = 0.046) had superior overall survival (OS), while GC patients with high expression of IL4R (HR 1.59, 95% CI 1.09–2.33, *p* = 0.016) and BMPR2 (HR 1.55, 95% CI 1.09–2.22, *p* = 0.015) had inferior OS (Fig. 8D). Consequently, we speculated that KPNA2 could regulate AS, which in turn affects the prognosis of patients with GC.

### Experimental verification

**Figure 9:**
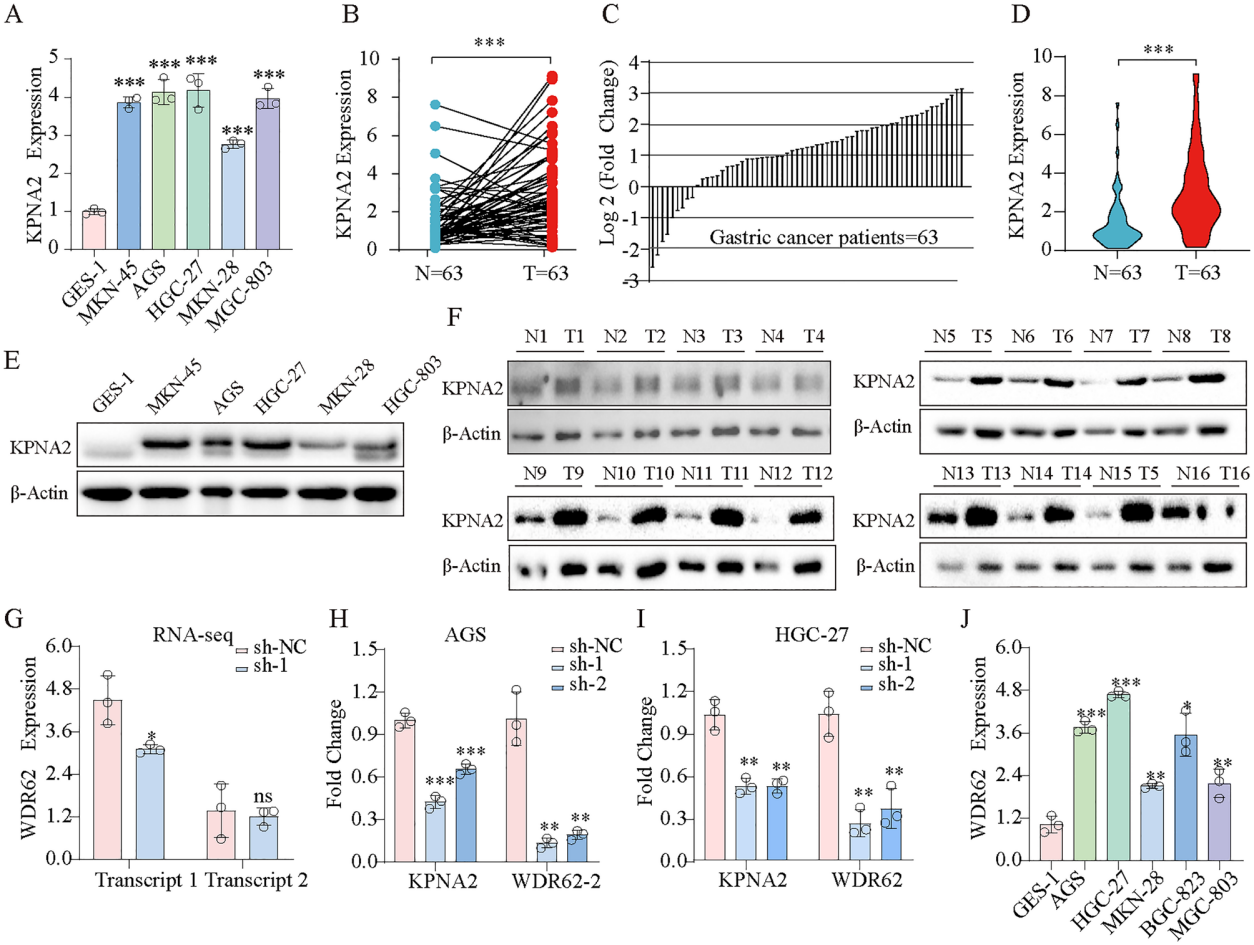
(**A**) Expression of KPNA2 mRNA in the normal GC cell line (GSE-1) and GC cell lines (MKN-45, AGS, HGC-27, MKN-28 and MGC-803). (**B**) Comparison of KPNA2 expression levels between 63 GC tissues and corresponding adjacent normal tissues. (**C**) Relative expression of KPNA2 in the 63 GC tissues and corresponding adjacent tissues. (**D**) Expression of KPNA2 in 63 pairs of GC tissues and corresponding adjacent tissues. (**E**) Expression of KPNA2 protein in the normal GC cell line (GSE-1) and GC cell lines (MKN-45, AGS, HGC-27, MKN-28 and MGC-803). (**F**) KPNA2 protein expression levels between GC tissues and corresponding adjacent normal tissues. (G-I) Downregulation of KPNA2 decreased WDR62 transcript 1 expression. (**G**) RNA-seq. (**H**) RT‒qPCR in AGS cell. (**I**) RT‒qPCR in HGC-27 cell. (**J**) Expression of WDR62 transcript 1 in the normal GC cell line (GSE-1) and GC cell lines (MKN-45, AGS, HGC-27, GCB-823, and MGC-803). *p < 0.05, **p < 0.01, ***p < 0.001.

Initially, we explored KPNA2 expression in GES-1, MKN-45, AGS, HGC-27, MKN-28, and MGC-803 cells and revealed that KPNA2 was dramatically upregulated in the GC cell lines (Fig. 9A). We then compared the expression of KPNA2 in GC tissues with that in corresponding adjacent GC tissues and evaluated the clinical significance of KPNA2 expression in 63 patients with GC. Forty-five patients with GC exhibited increased KPNA2 expression in cancer tissues (*p* < 0.0001, Fig. 9B), and 40 (63.5%) patients with GC exhibited KPNA2 mRNA levels that were at least twofold higher than those in the corresponding adjacent tissues (Fig. 9C). These results revealed that KPNA2 expression was significantly higher in GC tissues than in corresponding adjacent tissue (*p* < 0.001, Fig. 9D). And the protein level of KPNA2 was highly expressed in gastric cancer cells and gastric cancer tissues (Fig. 9E,F). Furthermore, we performed a Chi-fang analysis, which revealed that KPNA2 mRNA levels were correlated with lymph node metastasis (*p* = 0.001) and the Lauren classification (*p* = 0.000) but not with age, sex, tumor site, or pathological stage of patients with GC (*p* > 0.05, Table 3). RNA-seq revealed that the downregulation of KPNA2 decreased WDR62 transcript 1 (ENST00000401500.7) expression (Fig. 9G), and we verified this result by qRT‒PCR in AGS and HGC-27 cells (Fig. 9H,I). Moreover, WDR62 transcript 1 was highly expressed in GC cells (Fig. 9J).

**Table 3:** Clinicopathological characteristics of 63 GC patients from the first hospital of Lanzhou university.

Clinicopathological Feature	Number	KPNA2 expression		*p*
		Low	High	
Gender				
Male	48	26	22	0.237
Female	15	5	10	
Age (years)				
< 60	23	13	10	0.439
≥ 60	40	18	22	
T stage				
I–II	13	5	8	0.536
III–IV	50	26	24	
N stage				
N0–N1	25	6	19	**0.002**
N2–N3	38	25	13	
Pathologic stage				
Stage I–II	23	14	9	0.091
Stage III–IV	40	24	16	
Luauren classification				
Intestinal type	5	2	3	**0.000**
Diffuse type	22	14	8	
Mixed type	17	6	11	

Significant values are in bold.

**Figure 10:**
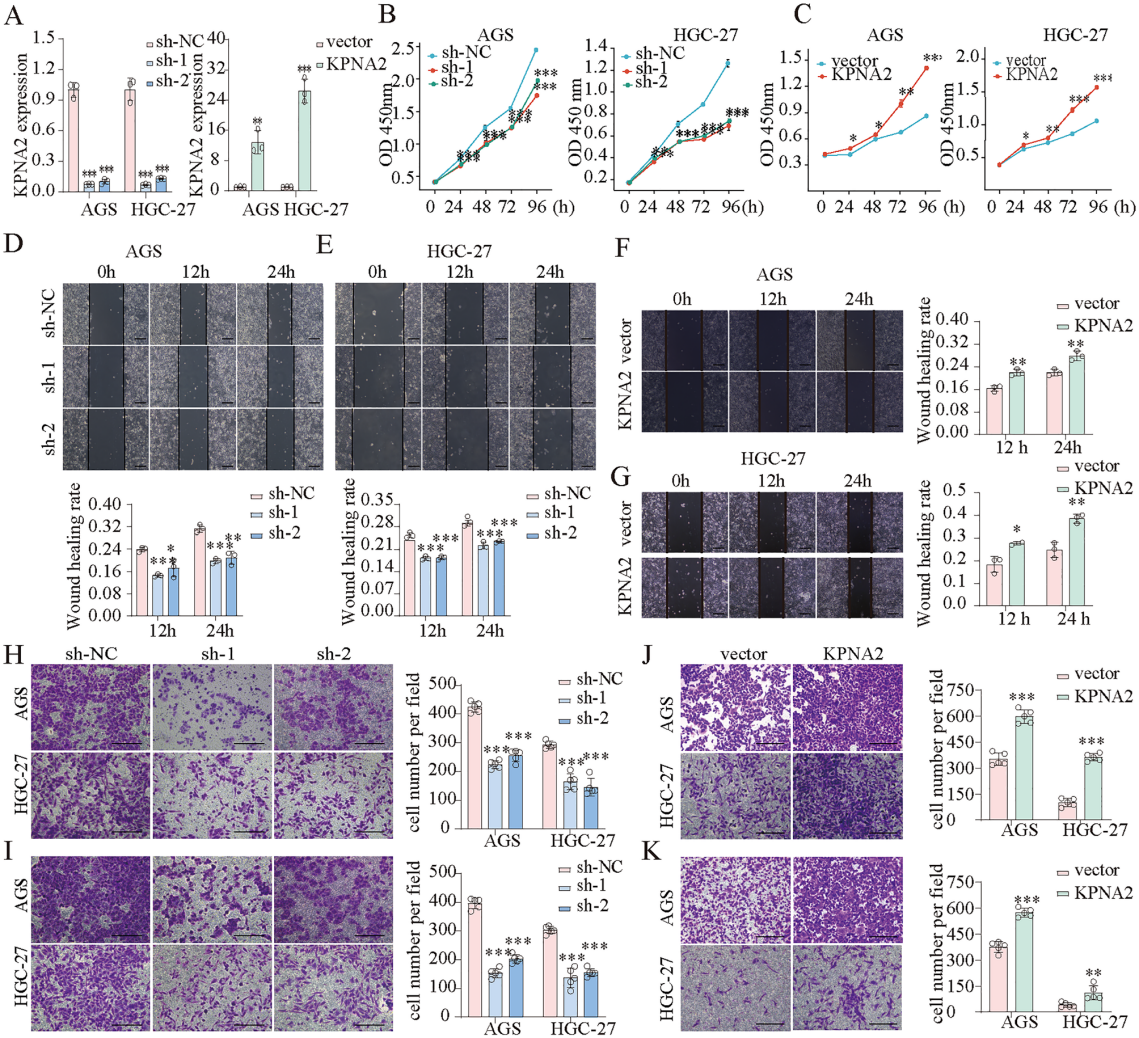
KPNA2 promoted the proliferation, migration and invasion of AGS and HGC-27 cells. (**A**) KPNA2 was downregulated or upregulated. (**B**,**C**) Cell proliferation was measured using CCK8 assay. (**D**–**G**) Cell migration was measured using a wound healing assay. (**H**–**K**) Cell migration and invasion were measured using Transwell assays. (**H**,**J**) Migration. (**I**,**K**) Invasion. *p < 0.05, **p < 0.01, ***p < 0.001.

**Figure 11:**
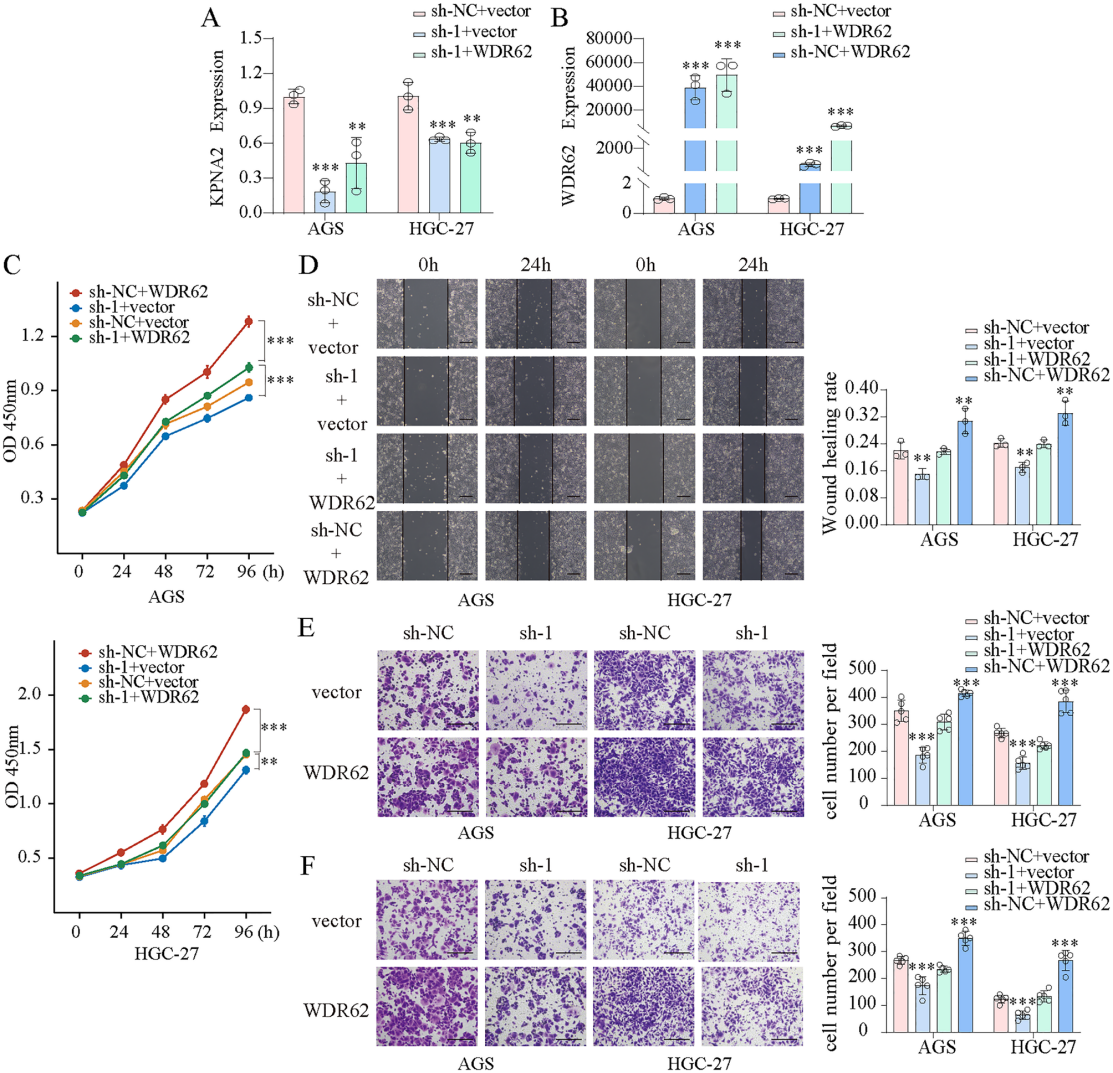
The oncogenic roles of KPNA2 in GC partially rely on WDR62 expression. (**A**,**B**) Downregulation of KPNA2 (sh-1) and upregulation of WDR62 in AGS and HGC-27 cells. (**C**) The potential of WDR62 to reverse the KPNA2-induced changes in proliferation (CCK8 assay). (**D**–**F**) The potential of WDR62 to reverse the KPNA2-induced changes in migration and invasion. (**D**) Wound healing assay, (**E**) Transwell migration assay, (**F**) Transwell invasion assay.

**Figure 12:**
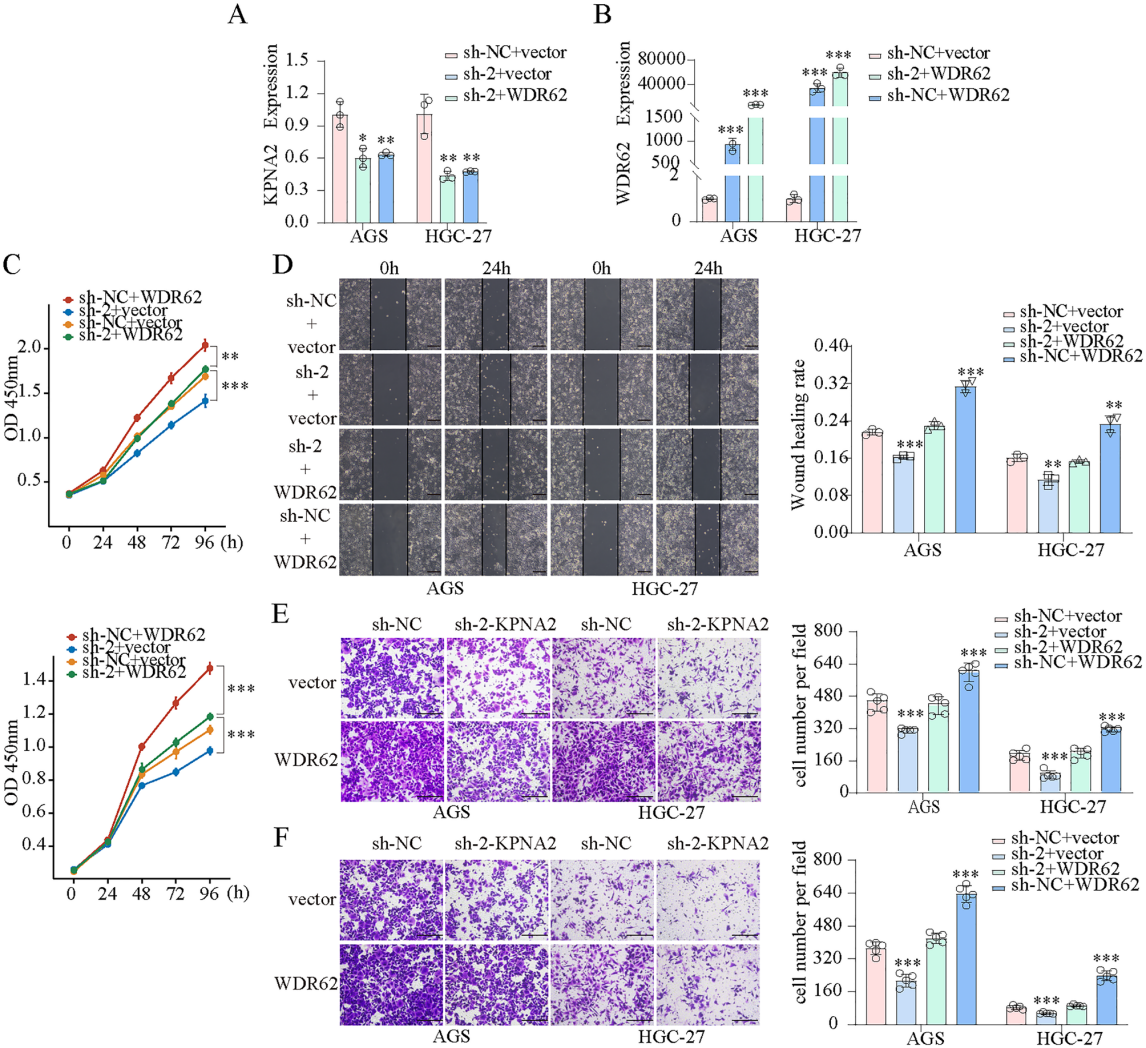
The oncogenic roles of KPNA2 in GC partially rely on WDR62 expression. (**A**,**B**) Downregulation of KPNA2 (sh-2) and upregulation of WDR62 in AGS and HGC-27 cells. (**C**) The potential of WDR62 to reverse the KPNA2-induced changes in proliferation (CCK8 assay). (**D**–**F**) The potential of WDR62 to reverse the KPNA2-induced changes in migration and invasion. (**D**) Wound healing assay, (**E**) Transwell migration assay, (**F**) Transwell invasion assay.

We transfected AGS and HGC-27 cells with KPNA2-shRNA and WDR62 plasmids and confirmed the transfection efficiency using qPCR (Fig. 10A). CCK8 assays showed that KPNA2 downregulation significantly inhibited the proliferation, whereas KPNA2 overexpression enhanced proliferation in AGS and HGC-27 cells (Fig. 10B,C). Wound healing and Transwell assays indicated that migration and invasion were significantly suppressed by KPNA2 downregulation or enhanced by KPNA2 overexpression in AGS and HGC-27 cells (Fig. 10D–K). Moreover, we simultaneously down-regulated KPNA2 (sh-1 or sh-2) and up-regulated WDR62 in gastric cancer cells (Figs. 11A,B, 12A,B), and found that overexpression of WDR62 reversed the KPNA2 knockdown-induced inhibition of proliferation (Figs. 11C, 12C), migration (Figs. 11D,E, 12D,E) and invasion (Figs. 11F, 12F). These results demonstrated that KPNA2 may play a pro-oncogenic role by regulating WDR62.

## Discussion

The TME contains a diverse range of cells, and infiltrating immune cells account for a large proportion of these cells^43^. In contrast to traditional view that considers immune cells to be part of an antitumor response, immune infiltration in the TME mirrors the immune escape of tumor cells^44,45^. Although KPNA2 has not been fully studied in the context of immuno-oncology, Th2 cells are known to be relevant to the establishment of an immunosuppressive environment and the induction of tumor immune escape^46^. Interestingly, one principal finding of our study was that KPNA2 expression was related to different levels of immune cell infiltration in GC, particularly immune cells that are associated with a suppressive immune context. The infiltration of Th2 cells was positively correlated with KPNA2 expression, and among all immune cells, the infiltration of these cells had the strongest association coefficient. As expected, the associations between KPNA2 expression and the expression of Th2 cell markers, such as NEIL3, CENPF, SNRPD1, and PHFR, remained the same as the general trend, suggesting that consistent mutual interactions occur between KPNA2 and certain Th2 cell subtypes. Subsequent RNA-seq revealed KPNA2 downregulation in AGS cells, changes in the expression of vast quantities of genes, and enrichment of pathways that were mostly associated with the immune response. Taken together, these findings suggest that KPNA2 is instrumental in the regulation of the TME by immune-infiltrating cells in GC, which may ultimately affect patient survival.

It has been established that abnormal AS events induce cancer^47^. Increasing evidence has also demonstrated that specific dysregulation of AS events plays a crucial role in the initiation and progression of GC^48^. RBPs can recognize and bind to exon or intron splice enhancer or silencer sequences in pre-mRNAs, facilitating or inhibiting the incorporation of the exon into the mature mRNA, respectively^7^. It has been demonstrated that splicing factors can regulate different AS events in different molecules; for example, DAP3 depletion promoted exon jumping in RBM6 but inhibited TIAL1 from undergoing A3SS^13^. Hence, we further explored the potential role of KPNA2 in regulating AS via RNA-seq. We first discovered that the AS levels of numerous genes were altered upon KPNA2 downregulation in AGS cells and that these gene-enriched pathways were mostly involved in the mitotic cell cycle. Increasing data suggest that the complexity of oncogenic networks increases owing to AS, which in turn promotes tumor progression^49,50^. Furthermore, using iRIP-seq, we discovered that cancer-associated mRNAs can bind to KPNA2. The iRIP-seq data demonstrated that the KPNA2-associated sequencing reads were mainly localized to the intron region, which provided new evidence that KPNA2 has functional RNA targets in intron regions and may participate in the transcriptional processing of RNA, which deserves further exploration. Furthermore, KPNA2 expression was associated with lymph node metastasis in our GC cohort. It has been reported that KPNA2, which is an important RBP, is involved in mRNA metabolic processes that promote cancer cell proliferation and invasion^51^. These data suggested that KPNA2 may regulate the AS of RNA by binding to mRNAs, which influences GC development.

By analyzing iRIP-seq and RNA-seq data, we found that the binding of KPNA2 to WDR62 pre-mRNA regulated the A3SS AS of WDR62. WDR62 plays an essential role in DNA replication and the cell cycle^52^. It has been reported that the proliferation of GC cells can be accelerated by WDR62^53^. The efficient splicing of pre-mRNAs is essential for the production of mature functional mRNAs^54^. We found that silencing KPNA2 inhibited WDR62 expression and that upregulation of WDR62 reversed the KPNA2 downregulation-induced inhibition of GC cell proliferation, migration and invasion. Taken together, these findings may elucidate how KPNA2 affects GC development.

The association between immunization and AS has emerged as a popular topic in the cancer field, but a limited set of patients who are responsive to immunotherapy show evidence of cancer-specific RNA splicing^55–57^. Researchers have observed that neoantigens that are generated by AS are widespread in most patients with cancer. These neoantigens may act as promising therapeutic targets for individuals with low tumor mutation loads^58^. We found that KPNA2 may regulate the AS of IRGs. Interestingly, the PI3K-AKT signaling pathway and transmembrane receptor protein serine/threonine kinase activity were analyzed to determine IPRG enrichment. Downstream signaling molecules, including AKT, can be recruited and activated through PI3K phosphorylation to increase T-cell effector function, and serine/threonine kinases can coordinate metabolic reprogramming to regulate T-cell differentiation and Treg cell function^59^. We also found that the prognosis of GC could be influenced by Co-IPRG (PPP4C, IL4R, and BMPR2); however, the exact mechanism involved needs to be further investigated.

In conclusion, KPNA2 is related to immune cell infiltration into GC, which may provide a new theoretical basis for antitumor immunotherapy based on the TME. Moreover, accumulating evidence has shown that AS plays an integral role in TME formation^60^. The regulation of AS by KPNA2 was demonstrated for the first time by the successful application of iRIP-seq and RNA-seq techniques in AGS cell lines. KPNA2 was shown to influence the immune response. Although direct regulation of AS between KPNA2 and WDR62 was identified, the exact mechanism by which KPNA2 regulates the AS of WDR62 has not been fully elucidated and requires further exploration. In summary, our work revealed new directions for targeted therapy of GC, and KPNA2 could serve as a novel prognostic biomarker and a prospective therapeutic target of GC.

### Supplementary Information

Supplementary Figure 1.

Supplementary Table 1.

Supplementary Table 2.

Supplementary Table 3.

Supplementary Table 4.

Supplementary Table 5.

Supplementary Table 6.

Supplementary Table 7.

Supplementary Table 8.

Supplementary Table 9.

Supplementary Table 10.

Supplementary Legends.

## Data Availability

We thank Wuhan Ruixing Biotechnology Co. for sequencing support. The data discussed in this publication are available under GEO Series accession number GSE201420.

## Code availability

The statistics and analysis of data were based on open-source software programming (R project, https://www.r-project.org/).
