# Supplementary figures and images for: KPNA2 promotes the progression of gastric cancer by regulating the alternative splicing of related genes
Source: Sci Rep. 2024 Jul 25;14:17140. doi: 10.1038/s41598-024-66678-7 (PMC11282077; doi:10.1038/s41598-024-66678-7)

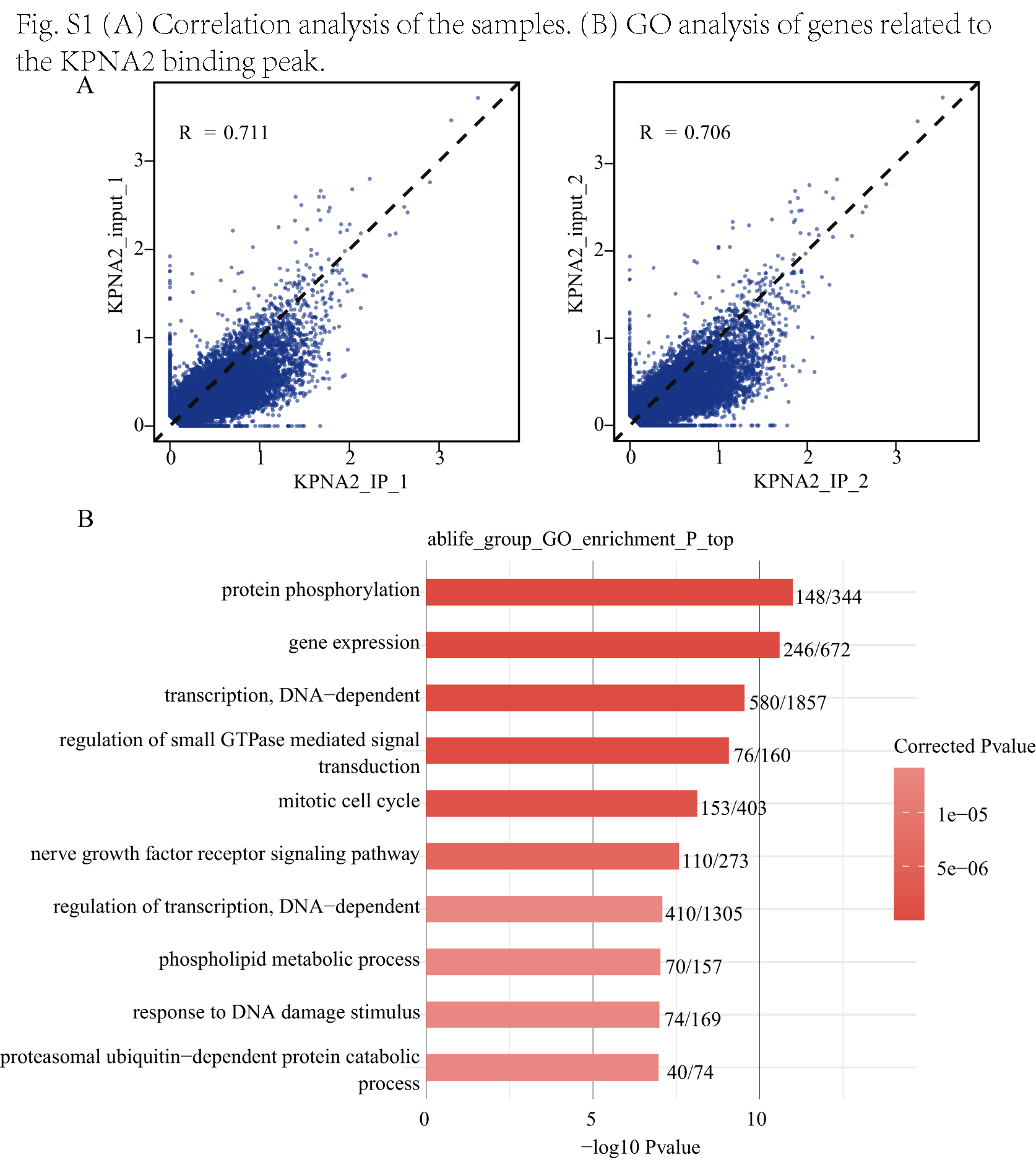

Supplement: Supplementary file 1 — Supplementary Figure 1. [file 41598_2024_66678_MOESM1_ESM.tif]
