# Supplementary material for: KPNA2 promotes the progression of gastric cancer by regulating the alternative splicing of related genes
Source: Sci Rep. 2024 Jul 25;14:17140. doi: 10.1038/s41598-024-66678-7 (PMC11282077; doi:10.1038/s41598-024-66678-7)
Supplement: Supplementary file 2 — Supplementary Table 1. [file 41598_2024_66678_MOESM2_ESM.docx]

| GAPDH-F | GGTCGGAGTCAACGGATTTG |
| --- | --- |
| GAPDH-R | GGAAGATGGTGATGGGATTTC |
| KPNA2-F | TGGAACAGTTGAACAGATTG |
| KPNA2-R | AACGAAGCCTTATACACAGA |
| WDR62-F | AGCTCTTCCCCGCAGC |
| WDR62-R | TCCAGGGTCTCTTCAGCCTT |
| ARID3A-F | CTCAGATGGCCGCACTGGCA |
| ARID3A-R | CGTCCTCCTCCTCCGAGCCTG |
| OAS3-F | CCAGCATATTCATAACTAGAGC |
| OAS3-R | GGAATGACTGCTAATGGGTT |
| SORT1-F | AGCTCACCCCGATGGGGCCAGAC |
| SORT1-R | ATGGCCTCGGCCTCCTCCTCCTC |
| TMEM14A-F | TCTGAGCATCTGGAGGAA |
| TMEM14A-R | GTTCATATTAGAGGTGACAGTG |
| TTYH2-F | CCTGTTGTCTCCAAGTAAGT |
| TTYH2-R | GGCCAATAAGCTCCTGTG |
| SERPINB2-F | TGCTCACTGCCTATTTAATG |
| SERPINB2-R | TGGAGGGATACCTACTTATTC |
| HPGD-F | TAAAGTGAGCAGAGGAAAGA |
| HPGD-R | GGGCAGTCAAGGAATAAAC |

Supplementary table1: Primer information
