# Supplementary material for: KPNA2 promotes the progression of gastric cancer by regulating the alternative splicing of related genes
Source: Sci Rep. 2024 Jul 25;14:17140. doi: 10.1038/s41598-024-66678-7 (PMC11282077; doi:10.1038/s41598-024-66678-7)
Supplement: Supplementary file 12 — Supplementary Legends. [file 41598_2024_66678_MOESM12_ESM.docx]

**Supplementary Table 1** Primer information

**Supplementary Table 2** Details of the DEGs regulated by KPNA2 in AGS cells

**Supplementary Table 3** Top 30 upregulated DEGs

**Supplementary Table 4** Top 30 downregulated DEGs

**Supplementary Table 5** Details of genes with changes in ASEs after KPNA2 downregulation

**Supplementary Table 6** Details of genes associated with the KPNA2 binding peak in the KPNA2-IP_1 group

**Supplementary Table 7** Details of genes associated with the KPNA2 binding peak in the KPNA2-IP_2 group

**Supplementary Table 8** Information on the 601 genes bound by KPNA2, which regulated their AS

**Supplementary Table 9** Details of WDHD1

**Supplementary Table 10** Information on the 17 KPNA2-binding IRGs
